# From Mild Cognitive Impairment (MCI) to Dementia in Chronic Obstructive Pulmonary Disease. Implications for Clinical Practice and Disease Management: A Mini-Review

**DOI:** 10.3389/fpsyg.2020.00337

**Published:** 2020-02-28

**Authors:** Laura Ranzini, Mara Schiavi, Antonia Pierobon, Nicolò Granata, Anna Giardini

**Affiliations:** Istituti Clinici Scientifici Maugeri IRCCS, Psychology Unit of Montescano Institute, Montescano, Italy

**Keywords:** cognitive impairment, Chronic Obstructive Pulmonary Disease, adherence, psychosocial factors, self-management, rehabilitation

## Abstract

Chronic Obstructive Pulmonary Disease (COPD) is a progressive disease characterized by partially irreversible chronic airflow limitation. Current literature highlights that COPD patients also have an increased risk to develop Mild Cognitive Impairment (MCI) and dementia. Chronic patients with cognitive impairment experience a worsening of health-related quality of life, mainly because it could affect treatment self-management, medication adherence and personal independence. Moreover, they also report high levels of anxiety and depression, which are associated with disease severity, poor quality of life, poor adherence to rehabilitation programs and difficulties in self-management. In current literature, there is a lack of studies describing simultaneously the associations between cognitive impairment, dysfunctional psychosocial factors, self-management abilities and their impact on pharmacological/non-pharmacological adherence. Therefore, the aim of the present short review is to describe the implications of cognitive impairment and psychosocial factors for clinical practice and disease management in COPD patients. Due to the interaction of these factors on adherence to rehabilitation programs, self-management and rehabilitation completion, future research should investigate simultaneously the role of all these different aspects to individuate a specific clinical approach that might include specific screening tools to evaluate cognitive impairment and psychosocial difficulties. A timely specific evaluation, within an interdisciplinary approach, could help to implement a more individualized and personalized treatment.

## Introduction

Chronic obstructive pulmonary disease (COPD) is a lung disease characterized by a partially irreversible chronic obstruction of lung airflow, with a typical onset after 55 years old (Singh et al., 2019). Several features of COPD (i.e., hypoxemia, hypercapnia, oxidative stress, and systemic inflammatory state) and comorbidities (i.e., cardiovascular disease), contribute to a higher risk of Mild Cognitive Impairment (MCI). In particular, patients with COPD may show cerebral perfusion alterations as a consequence of hypoxemia, which is an abnormal decrease in oxygen in the blood, and these changes could lead to cognitive deficits (Incalzi et al., 1993, 1997; Zheng et al., 2008; Dodd et al., 2010). It was demonstrated a 42% incidence of neuropsychological impairment in patients with hypoxemia and COPD as opposed to a 14% incidence in age matched controls (Grant et al., 1982, 1987). A further study underlined that 25% of people with COPD had MCI, while the overall prevalence of cognitive impairment for COPD was 32% (Yohannes et al., 2017).

Therefore, the aim of the present mini-review is to describe the implications of cognitive impairment and psychosocial factors for clinical practice and disease management in chronic diseases, with a specific focus on COPD patients.

## The Facets of Cognitive Decline

Dementia is characterized by multiple cognitive deficits, which progressively lead to impairment in activities of daily living, rapid cognitive decline, worse quality of life, earlier institutionalization, and greater caregiver depression (Prince et al., 2015).

For long time dementia was considered a natural problem related to the elderly, but in 1863 Nascher argued the thesis that dementia should be separated from physiological ageing which, on the other side, it is not pathological (Clarfield, 1990). Between the '90s and early 2000s, Petersen and colleagues introduced the term MCI referring to those patients who find themselves in an intermediate stage of cognitive impairment, that cannot be reported to normal cognitive aging and, at the same time, does not meet the criteria for the diagnosis of dementia (Petersen et al., 1999, 2001). Therefore, a consensus conference in 2003 led to the publication of international criteria for MCI, defined as a broader clinical syndrome with multiple subtypes due to a variety of etiologies (Winblad et al., 2004; Petersen and Morris, 2005). American Psychiatric Association [APA] (2013) has published new criteria for the diagnosis of the wide range of neurocognitive disorders in the fifth edition of the *Diagnostic and Statistical Manual of Mental Disorders* (DSM-5). The neurocognitive disorders are distinguished in Mild Cognitive Disorder (or better known as MCI) and Major Cognitive Disorder (or better known as Dementia) (American Psychiatric Association [APA], 2013). MCI and Dementia have been differentiated based on a combination of the period of time, the degree of impairment evaluated through standardized neuropsychological tests and the level of independence in the activities of daily living. Hence, the diagnosis is based primarily on cognitive, behavioral, and functional symptoms (Table 1).

**TABLE 1:** Clinical criteria for mild and/or major cognitive disorder diagnosis (American Psychiatric Association [APA], 2013).

**Mild cognitive disorder**	**Major cognitive disorder**

Cognitive decline from a previous level of performance in one or more cognitive domains: complex attention, executive function, learning and memory, language, perceptual motor, or social cognition	

A **modest** impairment in cognitive performance, preferably documented by standardized neuropsychological testing or, in its absence, another quantified clinical assessment.	A **substantial** impairment in cognitive performance, documented by a neuropsychological assessment.

The cognitive deficits **do not interfere** with capacity for independence in everyday activities	The cognitive deficits **interfere** with independence in everyday activities

The cognitive deficits are not better explained by another mental disorder and do not occur exclusively in the context of a delirium	

**FIGURE 1:**
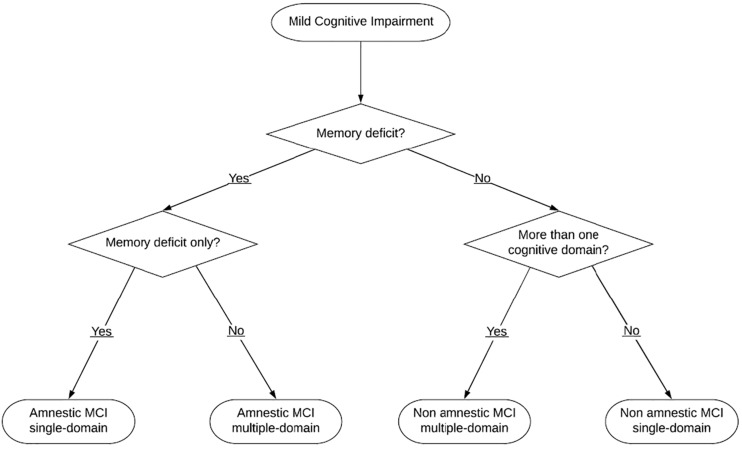
Clinical subtypes of MCI.

Petersen described clinical subtypes of MCI: non-amnestic MCI (na-MCI) and amnestic-MCI (aMCI), depending on whether or not memory is impaired. Furthermore, impairment could be referred to one cognitive domain (MCI single-domain) or multiple domains (MCI multiple-domains). In conclusion, four subtypes of MCI could be identified: aMCI single domain, aMCI multiple domains, na-MCI single domain and na-MCI multiple domains (Petersen and Morris, 2005; Figure 1).

The non-amnestic subtype in MCI is considered to be less common than the amnestic type (Petersen et al., 2010) and, while aMCI seems to represent an early stage of Alzheimer Disease, the outcomes of the na-MCI subtypes appear more heterogeneous, including vascular dementia, frontotemporal dementia or dementia with Lewy bodies (Mariani et al., 2007).

Another interesting study showed that although some patients could remain stable or return to normal cognition after being in a condition of MCI, they, however, still had a high risk of progressing to dementia. In particular, in a 5 years follow-up sample of 534 participants, 28.7% of subjects with MCI progressed to dementia while 38% of MCI participants reverted to normal cognition; however, 65% of the latter subsequently developed MCI or dementia. This suggests that making a diagnosis of MCI at any time has a prognostic value (Roberts et al., 2014).

Even if MCI definition refers to a syndrome that has no impact on the QQbasic activities of daily living (BADL) such as bathing, dressing or using the toilet, current literature suggests that MCI is characterized by a decrement in complex everyday tasks and instrumental activities of daily living (IADL). The compresence of cognitive and IADL impairments determine functional disability in everyday life and in disease management (Albert et al., 2009).

## The Importance of Evaluating Cognitive Decline in Chronic Patients

Literature highlights that people presenting one or multiple chronic conditions have a greater risk to develop MCI or dementia (Vassilaki et al., 2015). It is also known that chronic patients with MCI experience a worsening of Health-Related Quality of Life (HRQoL), an higher mortality and an higher incidence of rehospitalization than those without MCI (Ball et al., 2013; Lindbergh et al., 2016). Furthermore, the presence of these characteristics in chronic patients causes a burden on the caregivers in terms of care needed, time, fatigue and strain (Adelman et al., 2014). MCI patients exhibit difficulties in understanding complex clinical prescriptions and on judging the consequences of treatment choices (Okonkwo et al., 2008). The impaired capacity to plan, sequence and carry out tasks diminishes a patient’s ability to participate in decisions about their medical care and could determinate a higher risk for further serious clinical events. Medication non-adherence may result in toxicity due to altered pharmacodynamics both in MCI and in Dementia (Smith et al., 2017). Consequently, MCI and dementia are well-known risk factors for medication non-adherence, but also old chronic patients with initial cognitive decline may have difficulties in adherence to the prescribed treatments (Giardini et al., 2018). Therefore, it could be essential to have a time saving and specific instrument to evaluate cognitive functions in each different chronic disease, but, at the moment, scientific literature highlights that there are not adequate instruments for this purpose (Athilingam et al., 2015).

Particular interest deserve COPD patients since literature suggests that cognitive impairment is one of the most frequent comorbidities in COPD patients (Villeneuve et al., 2012; Fan and Meek, 2014; Pierobon et al., 2018) and this has a remarkable clinical and disease management relevance (Pierobon et al., 2017). In particular, both subtypes of MCI (amnestic and non-amnestic) could be detected in COPD patients (Singh et al., 2013), although the non-amnestic multiple domains MCI appears to be most frequent (Kakkera et al., 2018). Nevertheless, COPD is an underappreciated risk factor for dementia (Kakkera et al., 2018).

Furthermore, patients with COPD report high levels of anxiety and depression, which are associated with disease severity and are related to poorer quality of life, living alone, female sex, smoking, and lower socioeconomic status (Fan and Meek, 2014; Pierobon et al., 2017). Dulohery et al. (2015) confirm that living alone significantly affects the interaction between self-management abilities, COPD outcomes, and cognitive function. Moreover, depression and anxiety may adversely affect COPD self-management behaviors by decreasing adherence to pulmonary rehabilitation programs (Fan and Meek, 2014; Pierobon et al., 2017).

World Health Organization (WHO) stresses the importance of the interconnection between self-management and adherence in its own definition of adherence as “…the extent to which a person’s behavior – taking medication, following a diet, and/or executing lifestyle changes – corresponds with the agreed recommendations from a provider” (Sabaté, 2003). Finally, given the heterogeneity of the factors that are involved in COPD clinical course and the possible presence of comorbidities, such as MCI, it is of paramount importance to adopt an interdisciplinary approach to adequately evaluate and manage this clinical condition (Hillas et al., 2015).

## Impact of Cognitive Decline and Psychosocial Distress on Disease Management in COPD

Articles dealing with cognitive functioning and psychosocial factors on disease management and adherence in COPD were reviewed. To retrieve the articles included in the present mini-review, an electronic databases (PubMed, Medline, Scopus, PsycINFO) research was performed by using different combinations of keywords: *COPD, Chronic Obstructive Pulmonary Disease, chronic diseases, MCI, Mild Cognitive Impairment, cognitive impairment, dementia, adherence, compliance, self-care, self-management, daily activities, psychological, psychosocial.* After the electronic search was completed, the authors identified the eligible papers and the information collected was summarized and organized in a synoptic table (Supplementary Material).

As to disease management, Meek et al. (2001) in their longitudinal, study found that patients with COPD who have better cognitive function maintained the capacity to recall symptoms as dyspnoea and fatigue.

In a prospective study, Antonelli-Incalzi et al. (2008) showed that cognitive impairment might contribute to accelerate the decline of personal independence: patients with COPD and cognitive decline need more help in several basic and instrumental daily activities.

Dulohery et al. (2015) found no associations between cognitive function and self-management abilities or quality of life; however, the authors underlined that, despite normal cognitive functioning, there is a negative influence of living alone on self-management abilities (Dulohery et al., 2015).

Turan et al. (2017) in their cross-sectional study, outlined that cognitive impairment had negative consequences on disease self-management: lower cognitive functioning could affect the correct inhalation device technique. Furthermore, they underlined that socioeconomic status, smoking, pulmonary symptoms, and admission to hospital could have an effect on the adherence to inhalation therapy (Turan et al., 2017).

Greenlund et al. (2016) underlined that cognitive deficits, in particular confusion and memory loss, may affect functional limitations in COPD patients, increasing the need for assistance in different domains such as safety, transportation, house-hold activities, and personal care.

Furthermore, Baird et al. (2017) in their review article examined the impact of cognitive impairment on self-management in COPD patients and concluded that cognitive impairment in COPD increases the need for assistance in daily living, in treatment adherence and self-management (Baird et al., 2017).

Also, limited health literacy and deficits in fluid cognitive abilities contribute to low adherence, poor inhaler techniques and inadequate disease management (O’Conor et al., 2019).

Moreover, as to psychosocial factors, Fan and Meek (2014) analyzed the importance of both psychological symptoms (depression and anxiety) and cognitive impairment (working memory deficits) in affecting adherence to pulmonary rehabilitation programs: in particular, depression, anxiety and working memory deficits might adversely affect the completion of pulmonary rehabilitation. A high level of anxiety might also limit functional performance in the 6-minute walking test (Fan and Meek, 2014).

Finally, Pierobon et al. (2017) found that mild-severe depression and anxiety adversely affect adherence to exercise prescription at home in COPD patients. Lower depressive symptoms and high level of caregiver support lead to better disease management and adherence (Pierobon et al., 2017).

Currently, to the best of our knowledge, there is a lack of scientific literature describing simultaneously the associations between cognitive impairment, dysfunctional psychosocial factors, self-management abilities and their impact on pharmacological/non-pharmacological adherence.

## Conclusion

Evidence from current literature suggests that cognitive functioning should be always assessed in patients with a chronic disease, such as in COPD patients, mainly because different levels of cognitive impairment could affect self-management, adherence and personal independence. Moreover, recent articles on COPD underline the role of psychosocial factors, such as depression, anxiety and social support, on adherence to rehabilitation programs and self-management.

In COPD literature, cognitive impairment, psychosocial factors and their impact on self-management were considered mainly separated. It would be desirable, if not recommendable, that future researches investigate simultaneously all these different aspects due to their value and interconnection. About this issue, another aim for future research could be to individuate specific screening tools to evaluate cognitive impairment and psychosocial difficulties that should be tailored depending on the specific chronic condition considered.

As to clinical implications, besides clinical variables, patients’ adherence and self-management enhancement should focus both on psychosocial factors and cognitive status. Up to now, few evidence-based studies have allowed the identification of adequate intervention tools to increase adherence in chronic diseases (Costa et al., 2015).

Intervention on anxiety and depression should never be neglected since they could be useful to improve the patient’s emotional status and to indirectly intervene on adherence and self-management. Cognitive-behavioral therapy or psychological interventions could be implemented in the single-case situations where the presence of psychological difficulties could interfere with disease self-management, pulmonary rehabilitation adherence and outcome (Costa et al., 2017; Ma et al., 2019).

Moreover, given the multifaceted aspects of adherence and such a widespread presence of MCI or cognitive impairment in the COPD population, the rehabilitative project should be personalized and tailored to individual needs. It is clear that there are many factors to be considered and managed, thus an interdisciplinary approach should be always implemented to cope with this complex issue, in particular in inpatient and outpatient rehabilitation settings, in order to favor a more and more individualized and personalized treatment (Ambrosino and Clini, 2015).

## Author Contributions

LR and MS did the bibliographic search and wrote the first draft of the manuscript. AG and AP contributed to the conception and design of the study and with NG, they reviewed the first draft and wrote the final version of the manuscript. All authors contributed to manuscript review, read and approved the submitted version.

## Conflict of Interest

The authors declare that the research was conducted in the absence of any commercial or financial relationships that could be construed as a potential conflict of interest.
