# Supplementary material for: From Mild Cognitive Impairment (MCI) to Dementia in Chronic Obstructive Pulmonary Disease. Implications for Clinical Practice and Disease Management: A Mini-Review
Source: Front Psychol. 2020 Feb 28;11:337. doi: 10.3389/fpsyg.2020.00337 (PMC7058664; doi:10.3389/fpsyg.2020.00337)
Supplement: Supplementary file 1 [file Table_1.DOCX]

**Supplementary Material.** Articles reviewed dealing with cognitive functioning and psychosocial factors on disease management/adherence

| **Author (Year)** | **Study design** | **Sample Size** | **Cognitive functioning and/or psychosocial factors** | **Assessment tools** | **Impact on disease management, functionality and treatment adherence** |
| --- | --- | --- | --- | --- | --- |
| Meek et al. (2001) | Longitudinal descriptive study | 30 COPD patients | Cognitive functioning | - MMSE - Babcock story recall alternate form | The lower the cognitive function the greater the difference between the recalled symptom (dyspnea and fatigue) intensity and the actual value reported in the diaries. |
| Antonelli-Incalzi et al. (2007) | Prospective study | 149 COPD inpatients | Global cognitive functioning | - IADL - BADL - Visual-spatial intelligence - Phonemic verbal fluency - Visuospatial immediate memory - Short-term memory and attention - Visual exploration - Verbal memory - Constructional abilities - Verbal competence | The study suggest that cognitive impairment might contribute to speed the decline of personal independence: low level of cognitive functioning correlates with high level of external help in several basic and instrumental daily activities |
| Fan et al. (2014) | Review | - | Depression, anxiety, cognitive impairment | - | Psychological symptoms working memory deficits may affect adherence to pulmonary rehabilitation programs |
| Dulohery et al. (2015) | Cross-sectional study | 100 COPD patients | Cognitive impairment, HRQoL, Depression | - MoCA - SMAS-30 - General survey - Pulmonary function testing - 4-meter gait speed test - PHQ-2 - CRQ - mMRC Dyspnea scale - ADO index | No association between cognitive function and important COPD outcomes (exacerbation, emergency room visit or hospitalization). No association between cognitive function and self-management abilities or quality of life; only patient living alone with higher cognitive function report lower self-management abilities. |
| Turan et al. (2016) | Cross-sectional study | 88 COPD patients | Global cognitive functioning, HRQoL | - MMSE MMAS-4 - SGRQ - Self report about inhalation therapy | Cognitive impairment can affect inhalation device technique. Socioeconomic status, smoking, pulmonary symptoms and admission to hospital because of airway disease were also thought to have effect on the adherence to inhalation therapy. |
| Greenlund et al. (2016) | State-based telephone survey | 10476 COPD patients | Qualitative evaluation of cognitive functioning | - Behavioral risk factor Surveillance System, - Self-report instrument that assess health status, confusion or memory loss and limitation in daily activities | Increased confusion and memory loss may affect functional limitation in COPD patients: from one-third to two-thirds of patients reported to need assistance in at least 1 of 5 domains (safety, transportation, house-hold activities, personal care or some other domain) |
| Baird et al. (2017) | Review | - | Cognitive impairment/ dementia | - | Cognitive impairment in COPD increase the need for assistance in daily living, in treatment adherence and self-management |
| Pierobon et al. (2017) | Multicentre observational cross-sectional study | 84 COPD inpatients | MCI, depression. anxiety | - MMSE - MoCA - HADS-A - BDI-II - ASiCOLD-R - BMI | Depression mild-severe and anxiety independently predict lower adherence to exercise prescription at home. Lower depressive symptoms and higher familial support, less frequent LTOT modification are present. |
| O’Conor et al. (2019) | Observational cohort study | 393 COPD patients (333 after follow-up at 12 months; 299 after follow-up at 24 months) | Global cognitive functioning | - MARS - Validated standardized checklist about inhaler technique - S-TOFHLA - MMSE - Processing speed - Working memory - Long-term memory - Executive functions - Semantic memory | COPD patients with limited health literacy and deficits in fluid cognitive abilities have lower rates of adherence and poorer inhaler techniques than individuals with adequate health literacy and greater fluid cognitive abilities. |

Abbreviations: ADO, Age Dyspnea Obstruction index; ASiCOLD-R, Adherence Schedule in Chronic Obstructive Lung Diseases-Revised; BADL, Basic Activities of Daily Living; BDI, Beck Depression Inventory; BMI, Body Mass Index; CRQ, Chronic Respiratory Disease Questionnaire; HADS-A, Hospital Anxiety and Depression Scale-Anxiety; HRQoL, Health Related Quality of Life; IADL, Instrumental Activities of Daily Living; MARS, Medication Adherence Reporting Scale; MMAS, Morisky Medication Adherence Scale; mMRC, modified Medical Research Council Dyspnea scale; MMSE, Mini Mental State Examination; Moca, Montreal Cognitive Assessment; PHQ-2, Patient Health Questionnaire; SGRQ, St. George’ Respiratory Questionnaire; SMAS-30, Self-Management Ability Scale; S-TOHFLA, Short Test of Functional Health Literacy in Adults.
